# Network Pharmacological Analysis of Hydroxychloroquine Intervention in the Treatment of Iga Nephropathy

**DOI:** 10.2174/0113816128347345241028063515

**Published:** 2024-11-01

**Authors:** Mengxiao Zou, Gang Xu, Shuwang Ge, Kanglin Guo, Qian Duo, Yichun Cheng

**Affiliations:** 1 Division of Internal Medicine, Department of Nephrology, Tongji Medical College, Tongji Hospital, Huazhong University of Science and Technology, Wuhan 430030, China

**Keywords:** Hydroxychloroquine, IgA nephropathy, network pharmacology, toll-like receptor, signaling pathway, end-stage renal disease

## Abstract

**Background:**

IgA nephropathy (IgAN) is the most prevalent primary glomerulonephritis globally and has a high propensity to develop into end-stage renal disease (ESRD). Hydroxychloroquine has been proven to reduce proteinuria in IgAN patients, but the precise mechanism remains unclear. Therefore, network pharmacology was used to investigate the mechanism.

**Methods:**

PubChem and SwissADME databases were utilized to acquire the structure of hydroxychloroquine. The SwissTargetPrediction, PharmMapper, DrugBank, TargetNet, and BATMAN-TCM databases were then utilized to obtain the targets. The target genes related to IgAN were then gathered from the databases, which included GeneCards, PHARMGKB, DrugBank, OMIM, and DisGeNET. Common targets were obtained by UniProt. Gene Ontology (GO) and Kyoto Encyclopedia of Genes and Genomes (KEGG) enrichment analyses were performed to define the main molecular mechanisms and pathways. Furthermore, a protein-protein interaction (PPI) network was constructed using the STRING tool, and the core targets were obtained by Cytoscape. Finally, molecular docking between the core targets and hydroxychloroquine was performed.

**Results:**

167 common target genes were acquired by overlapping. The core targets were TNF, ALB, IL1B, JUN, FOS, SRC, and MMP9. The GO and KEGG results showed the targets to be related to the production of inflammatory cytokines and chemokines and were engaged in the toll-like receptor (TLR) signaling pathway. At the same time, the molecular docking results showed that the core targets all combined with hydroxychloroquine closely.

**Conclusion:**

This study proved that hydroxychloroquine may treat IgAN through the TLR signaling pathway, and the restraint of TNF, TLR, IL1B, and JUN may be essential for the treatment.

## 1 INTRODUCTION

IgA nephropathy (IgAN) is a primary glomerular disease that has the highest prevalence in the world [1-3], particularly in Asia [4]. It is characterized by the occurrence of IgA-dominant or co-dominant immune deposits in the glomerulus [5]. Approximately 30-40% of patients with IgA nephropathy in China rapidly progress to end-stage renal disease (ESRD) within 10 years due to the lack of exact treatments [6-8]. Although some studies have proposed a “four-step attack” hypothesis as a means of explaining the process of renal damage [9], it cannot fully explain the pathogenesis of IgA nephropathy. This may be why there is currently no effective drug for the treatment of IgA nephropathy. The major goals of IgA nephropathy treatment are lowering blood pressure, conserving renal function, and reducing proteinuria. The main medications that are currently used for the treatment of IgA nephropathy include chronic activation of the renin-angiotensin-aldosterone system (RAAS) blockers, glucocorticoids, and immunosuppressants [10, 11]. However, the benefit of RAAS blockers in reducing albuminuria is limited; glucocorticoids often have severe side effects, and the clinical manifestations of immunosuppressants are unstable [12]. At the same time, some studies have found the addition of hydroxychloroquine to RAAS inhibitors to be effective for relieving proteinuria with a low incidence of side effects [13].

Hydroxychloroquine is an anti-malarial drug with immunomodulatory and anti-inflammatory effects that previous studies have proven to delay renal damage in autoimmune disease treatments, including systemic lupus erythematosus and rheumatoid arthritis [14-17]. Studies have also found hydroxychloroquine to be more beneficial for relieving proteinuria and stabilizing renal function in IgA nephropathy than other methods [18, 19]. However, the mechanism of hydroxychloroquine in IgA nephropathy treatment remains unclear.

Network pharmacology (NP) is an effective way of discovering new drugs and studying the mechanism between diseases and drugs through the construction of a network of “drug components-action targets-signal pathways-action mechanisms-disease” [20-22]. NP describes the complex relationship among drugs, target proteins, and diseases from a network perspective while also conducting multi-target studies. Mechanisms and targets for hydroxychloroquine in the treatment of systemic lupus erythematosus (SLE) through network pharmacology have been identified by several studies [20, 23]. However, the pathogenesis of IgAN and SLE are different, and the targets of hydroxychloroquine for IgAN therapy may be different, so further studies are required.

This study, we used NP for exploring the mechanism of hydroxychloroquine for IgA nephropathy treatment. The targets of hydroxychloroquine and IgA nephropathy were overlapped to obtain common targets, PPI networks for the common targets were constructed to find their interactions, the GO and KEGG results of the common targets were analyzed, and they were ultimately validated by molecular docking.

## 2 MATERIALS AND METHODS

### 2.1 Chemical Structure and Drug Properties of Hydroxychloroquine

The PubChem ID [24], 3D structure, 2D structure, international compound identification (InChI), and standard simplified molecular input line entry system (SMILES) of hydroxychloroquine were acquired from the PubChem database. The drug similarity and gastrointestinal absorbance of hydroxychloroquine were obtained from the SwissADME database [20].

### 2.2 Targets Protein of Hydroxychloroquine

Taking humans as the target species, hydroxychloroquine target proteins were predicted from the SwissTargetPrediction database [25], PharmMapper database [26], DrugBank database [27], TargetNet database [28], and BATMAN-TCM database [29].

### 2.3 Target Protein of IgAN

Taking humans as the target species, IgA nephropathy target proteins were acquired from the GeneCards database [30], PHARMGKB database [31], DrugBank database, OMIM database [32], and DisGeNET database [33].

### 2.4 Common Targets of Hydroxychloroquine and IgA Nephropathy

IgA nephropathy target protein and hydroxychloroquine target protein were verified by UniProtKB ID [34]. The collected target protein information was then standardized in order to obtain the corresponding genes and the common target genes.

### 2.5 PPI Network

The PPI network on common target genes was constructed through the STRING database [35], according to the lowest interaction score (maximum > 0.9; high > 0.7; medium > 0.4; low > 0.15). 0.4 (medium) was set as the lowest interaction score in this study. Cytoscape was used to build a clearer PPI network [36]. The larger the value of the target, the darker the color, the larger the area, and the closer to the center of the circle.

### 2.6 Core Network Construction

Cytoscape-CytoNCA was used to obtain the degree of betweenness centrality, and closeness centrality of each node in the network. The network was filtered with the average of the three parameters as the minimum to create a sub-network. Similarly, the core network of PPI was constructed.

### 2.7 GO and KEGG Enrichment Analysis

The species was selected as humans, the common targets were analyzed by the Metascape database to get the GO and KEGG enrichment analyses results [37], and *p* < 0.01 was set as statistically significant. Following the analyses, the top 20 items of biological processes (BP), cellular components (CC), molecular function (MF), and KEGG were chosen for visualization. According to the common targets related to each pathway, Cytoscape was used to analyze, and a target-pathway network was constructed.

### 2.8 Molecular Docking

ChemBio3DUltra was used to draw the 3D chemical structure of hydroxychloroquine. Crystal structures of target proteins were obtained by querying the RCSB-PDB database [38], and SYBYL was used to dehydrate and hydrogenate the target proteins for the structural domain. They were then combined with hydroxychloroquine to obtain the CSCORE.

### 2.9 Statistical Analysis

All statistical data were analyzed with the use of R, version 4.0.5. Statistical significance was defined as *p* < 0.05.

## 3 RESULTS

### 3.1 Chemical Structure and Drug Properties of Hydroxychloroquine

The chemical structure information of hydroxychloroquine was obtained (Table **1**).

### 3.2 Target Proteins of Hydroxychloroquine and IgA Nephropathy

Six hundred eleven target proteins of hydroxychloroquine were identified by searching the DrugBank database, TargetNet database, PharmMapper database, SwissTargetPrediction database, and BATMANTCM database. IgA nephropathy-related target proteins were searched for in five databases. After merging, one thousand four hundred and sixty-one target proteins of IgA nephropathy were collected, and the target genes that corresponded to the above target proteins were obtained from the UniProt database. Detailed information is shown in Tables **2** and **3**.

### 3.3 PPI Network Common Targets for Constructing a Core Target Network

**Fig. (1):**
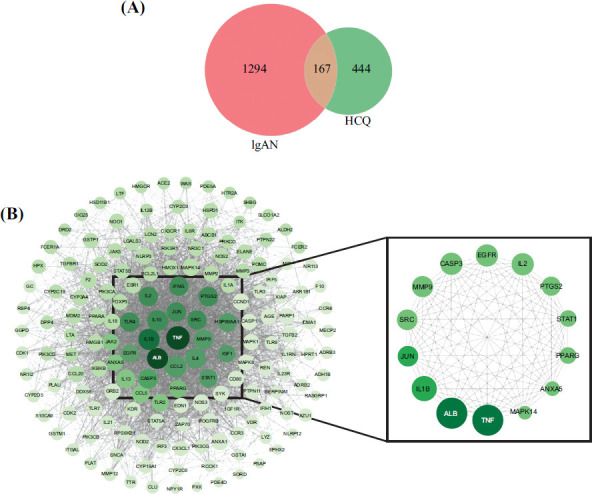
(**A**) Common targets of hydroxychloroquine and IgAN; (**B**) PPI network and core network.

One hundred sixty-seven common target genes may be prospective hydroxychloroquine targets in IgAN therapy (Fig. **1A**). A PPI network and the core network were constructed using the common targets. The core network consisted of 14 nodes and 91 edges. The degree value, dielectric value, and tightness value of the 14 nodes in the core network can be seen in Table **4**, and the above three parameters of TNF, ALB, and IL1B were all found to be higher than average (Fig. **1B**).

### 3.4 GO Enrichment Analysis and KEGG Pathway Enrichment Analysis

**Fig. (2):**
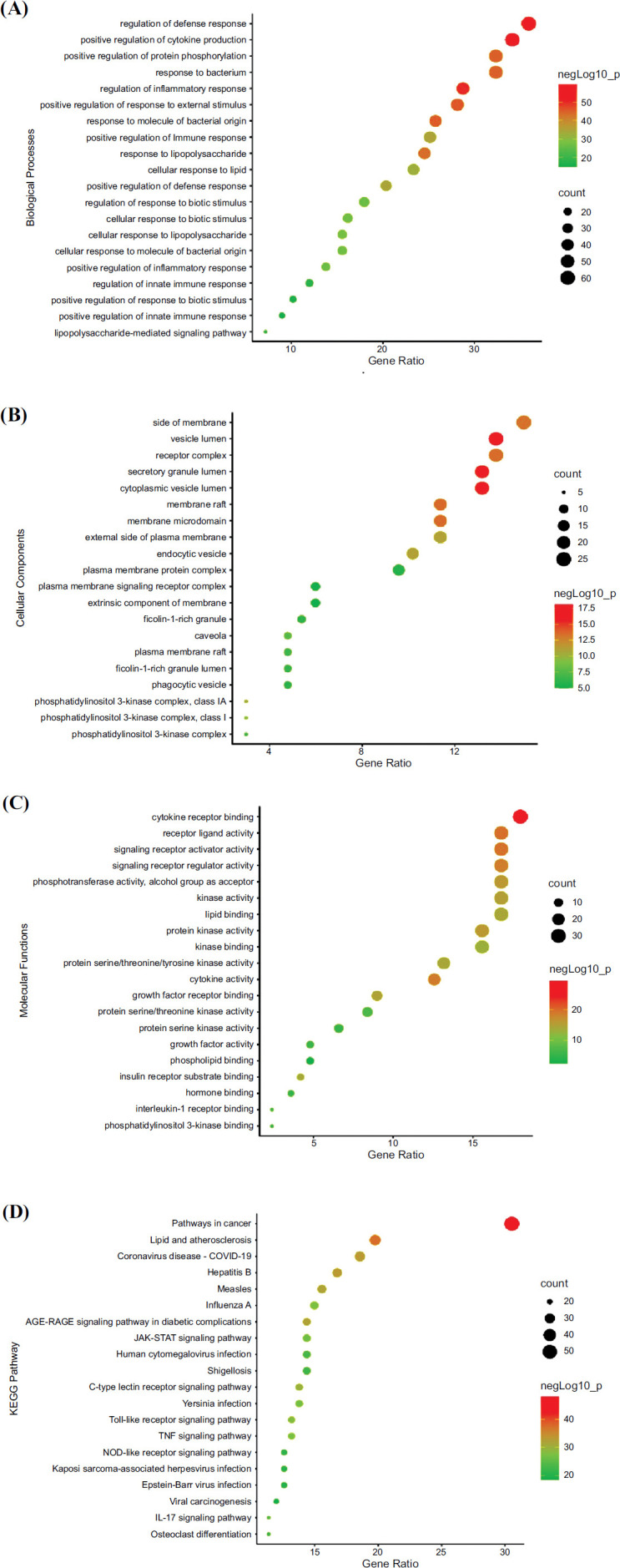
(**A**) Biological processes of GO analysis; (**B**) Molecular functions of GO analysis; (**C**) Cell components of GO analysis; (**D**) KEGG pathway enrichment analysis.

GO and KEGG enrichment analyses were conducted on the hydroxychloroquine targets of IgA nephropathy therapy. One thousand nine hundred fifteen biological processes were scrutinized, which included cytokine production, defense response, inflammatory response, and immune response (Fig. **2A**). One hundred eighty-two molecular functions were obtained, which included receptor-ligand activity, signaling receptor activator activity, cytokine activity, and signaling receptor regulator activity (Fig. **2B**). Seventy-five cell components were investigated and these mainly included various cell structures: veslumen, secretory granule lumen, cytoplasmic vesicle lumen, ficolin-1-rich granule lumen, Ficolin-1-rich granule (Fig. **2C**).

One hundred sixty-seven main signal pathways were obtained from KEGG enrichment, mainly including pathways in cancer, JAK-STAT signaling pathway, lipid and atherosclerosis, hepatitis B, Coronavirus disease - COVID-19, and toll-like receptor signaling pathway. Of the 20 pathways, lipid and atherosclerosis, JAK-STAT signaling pathway, AGE-RAGE signaling pathway, and toll-like receptor signaling pathway may be related to the potential mechanism of hydroxychloroquine for IgAN treatment (Fig. **2D**).

### 3.5 The Crystal Structures of Target Proteins and Molecular Docking

The top 10 targets in the core network -TNF, ALB, IL1B, JUN, SRC, MMP9, CASP3, EGFR, IL2, and PTGS2 - and the targets in the toll-like receptor signaling pathway - TLR2, TLR3, and TLR4 - were selected for molecular docking. Their corresponding crystal structures were acquired from the Unispot database.

Table **5** shows the molecular docking results. CSCORE evaluates the docking effect between molecules and ranges from 0 to 5. A higher value means the docking effect is better and that the molecular docking is closer. The results suggested IL1B, JUN, TLR2, TLR3, and TLR4 all had extremely high binding activity with hydroxychloroquine. ALB, EGFR, IL2, MMP9, PTGS2, SRC, and TNF were all found to have high combined activity with hydroxychloroquine.

The target proteins of the toll-like receptor signaling pathway, including IL1B, JUN, TLR2, TLR3, TLR4, and TNF, all combine with hydroxychloroquine tightly.

**Fig. (3):**
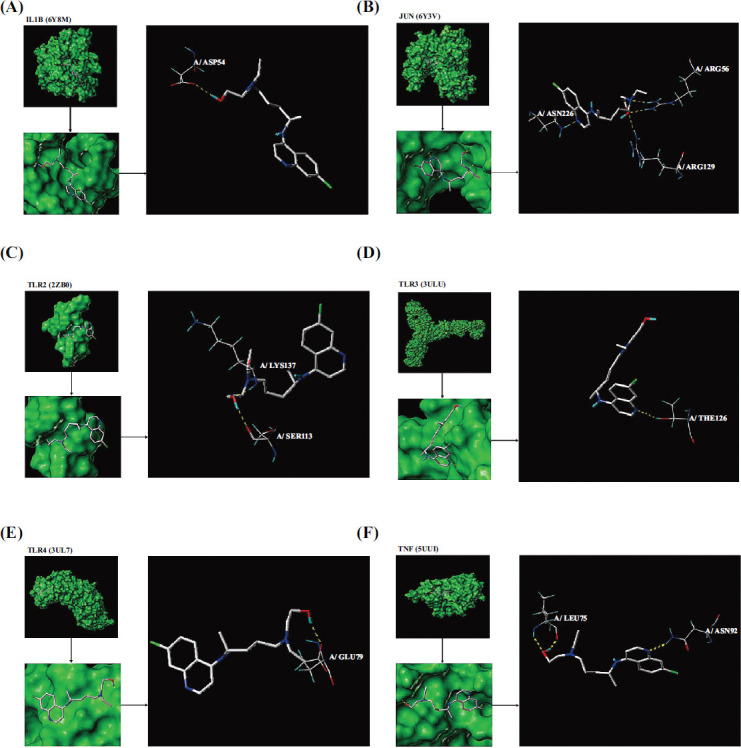
(**A**) Hydroxychloroquine bound to IL1B; (**B**) Hydroxychloroquine bound to JUN; (**C**) Hydroxychloroquine bound to TLR2; (**D**) Hydroxychloroquine bound to TLR3; (**E**) Hydroxychloroquine bound to TLR4; (**F**) Hydroxychloroquine bound to TNF.

Hydroxychloroquine bound to one amino acid site of IL1B, A/ASP54, by a hydrogen bond (Fig. **3A**). Hydroxychloroquine bound to three amino acid sites of JUN, A/ARG56, A/ARG129, and A/ASN226 by a hydrogen bond (Fig. **3B**). Hydroxychloroquine bound to two amino acid sites of TLR2, A/LYS137 and A/SER113, by a hydrogen bond (Fig. **3C**). Hydroxychloroquine bound to one amino acid site of TLR3, and A/THR126, respectively, by a hydrogen bond (Fig. **3D**). Hydroxychloroquine bound to the A/GLU79 amino acid site of TLR4 by a hydrogen bond (Fig. **3E**). Hydroxychloroquine bound to two amino acid sites of TNF, A/LEU75, and A/ASN92, by a hydrogen bond (Fig. **3F**).

## 4 DISCUSSION

One hundred sixty-seven common targets were obtained in this study by merging the targets of hydroxychloroquine and IgAN. The core network had 14 nodes: TNF, ALB, IL1B, JUN, SRC, MMP9, CASP3, EGFR, IL2, PTGS2, STA T1, PPARG, ANXA5, and MAPK14. The results of the GO analysis and KEGG enrichment analysis suggested that the generation of different inflammatory cytokines and chemokines through the toll-like receptor signaling pathway is primarily controlled by hydroxychloroquine. The molecular docking results indicated that IL1B, JUN, ALB, EGFR, IL2, MMP9, PTGS2, SRC, TNF, TLR2, TLR3, and TLR4 all had high binding activity with hydroxychloroquine. Of these targets, IL1B, JUN, TLR2, TLR3, TLR4, and TNF were found in the Toll-like receptor signaling pathway, meaning that the TLR signaling pathway could be important in the process of the treatment of IgAN by hydroxychloroquine.

The core targets in PPI were TNF, ALB, IL1B, JUN, FOS, SRC, and MMP9. ALB regulates the plasma colloid osmotic pressure and participates in the of cell apoptosis regulation [39]. Low serum albumin is a risk factor for adverse IgA nephropathy outcomes [40]. TNF is a key cytokine that is related to apoptosis, cell survival, inflammatory response, and immune regulation [41, 42]. The supernatant suspensions that contain IgA1 from IgA nephropathy patients will promote the expression of TNF and its receptors by human mesangial cells [43] and glomerular hyperpermeability and proteinuria will be caused. TNF expression in IgAN patients is higher than among normal people [44], and its activation is closely related to renal fibrosis [45]. Rahman *et al*. discovered that hydroxychloroquine could significantly reduce the generation of TNF-α [46]. IL1B belongs to the IL1 family [47, 48], which is a powerful regulator of inflammation that is closely linked to mesangial cell proliferation and extracellular matrix production [49]. IL1B is produced locally in the glomeruli and mesenchyme in IgA nephropathy, leading to ongoing renal injury [50, 51]. Some studies found that IL1B expression can potentially be influenced by IgA concentration [52]. JUN and FOS are members of the transcription factor AP1, which participates in proliferation, cell death, differentiation, and inflammation [53, 54]. A precise bioinformatic analysis has found that JUN and FOS play significant roles in IgAN fibrosis progression [55]. SRC is involved in signaling pathways that regulate a wide range of biological activities, including gene transcription, immune response, cell adhesion, cell cycle progression, apoptosis, migration, and differentiation [56, 57]. SRC kinase inhibitor usage has been found to prevent renal fibrosis in mice [58]. MMP9 is a member of the MMPs, and it can degrade and decompose glomerular extracellular matrix proteins. It is essential in renal disease development. MMP9 is secreted by glomerular thylakoid cells and enhanced MMP9 activity stimulates glomerular matrix degradation, which contributes to glomerular structure and function changes [59]. An experimental study found the development of kidney diseases such as hypertensive glomerulosclerosis and diabetic nephropathy to have a correlation with the downregulation of MMP9 [60].

The GO and KEGG analysis results indicated that the common targets are primarily involved in the inhibition of cytokines, inflammation, and immune response. IgA nephropathy is a multifactorial disease that is related to chronic inflammation [11]. Numerous previous studies have found inflammation to be the key factor that drives IgA nephropathy occurrence and development [4, 61, 62]. At the same time, an abundance of inflammatory cytokines and cytokine receptors in the common target may be engaged in the cancer pathway, including TNF, IL6, and IL2. This potentially explains why the cancer pathway ranks quite high in KEGG analysis.

The molecular docking results showed that IL1B, JUN, TLR2, TLR3, TLR4, and TNF in TLR signals all have high binding activity to hydroxychloroquine, thereby confirming that hydroxychloroquine may inhibit immune function by blocking the transduction of the TLR signal pathway.

TLRs are members of non-specific immune receptors that are expressed in the membranes of dermal cells and renal tubular epithelial cells. They induce inflammatory cytokine expressions through intracellular signaling pathways [63-65]. Activated TLRs can directly damage the kidney and produce excessive antibodies through B lymphocytes [66]. TLR2 activation can induce renal tubulointerstitial inflammation and increase proteinuria in mice [67]. TLR4 may regulate the concentration and glycosylation level of IgA1 through its participation in NF- κB activation [68]. At the same time, TLR4 expression will be up-regulated by podocytes that respond to immune complex-mediated glomerular filtration barrier injury. This may lead to the local release of chemokines and the absorption of inflammatory leukocytes, in addition to aggravating glomerular injury [69]. Zou *et al*. used a mouse model of IgA nephropathy as a means of proving that inflammatory response and TLR4 signaling pathway are related to IgA nephropathy progression [70, 71]. Han *et al*. and Sato *et al*. suggested that hydroxychloroquine can inhibit TLRs to reduce inflammatory cytokines production and thus treat autoimmune diseases [72, 73]. Therefore, hydroxychloroquine may inhibit the progression of IgA nephropathy by blocking the signal transduction of the TLR signal pathway and inhibiting inflammatory cytokines production.

This study used NP as a means of preliminarily predicting the potential target sites and pathways of hydroxychloroquine in IgAN treatment and provided a reference for the clinical treatments and new drugs of IgA nephropathy. Targets were collected from several public and reliable databases to ensure the diversity of targets. However, the renewal cycle of databases will affect the accuracy of the results. This study is only a prediction model and cannot serve to replace experiments on the relationship between drugs and molecular targets. Future animal experiments and cell experiments are required in order to confirm the mechanism.

## CONCLUSION

This study explored the key targets, signal pathways, and mechanism of hydroxychloroquine in IgA nephropathy treatment through NP, verified by molecular docking. It was indicated that hydroxychloroquine may delay IgA nephropathy progression by interfering with the signal transduction of toll-like receptor signal transduction and reducing inflammatory cytokines production.

## Figures and Tables

**Table 1:** Chemical structure of hydroxychloroquine.

**Hydroxychloroquine**	
**PubChem ID**	**3652**
3D structure	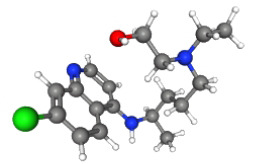
2D structure	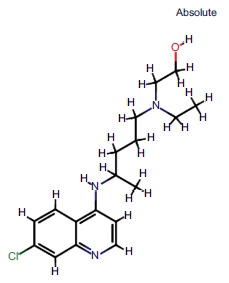
InChI	InChI=1S/C18H26ClN3O/c1-4-22(11-14-23)10-4-5-14(2)21-17-8-9-20-18-14-15(19)6-7-16(17)18/h6-9,14-14,23H,4-5,10-12H2,1-2H3,(H,20,21)
Standard SMILES	CCN(CCCC(C)NC1=C2C=CC(=CC2=NC=C1)Cl)CCO

**Table 2:** Target proteins associated with hydroxychloroquine.

**Database**	**Number of Target Proteins**
DrugBank database	11
TargetNet database	27
PharmMapper database	291
SwissTargetPrediction database	115
BATMAN-TCM database	251
Summary	611

**Table 3:** Target proteins associated with IgA nephropathy.

**Database**	**Number of Target Proteins**
DrugBank database	20
GeneCards database database	1066
OMIM database	173
PHARMGKB database	107
DisGeNET database	456
Summary	1461

**Table 4:** The information on common targets in the core network.

**Gene Symbol**	**Name of Proteins**	**Degree Value**	**Dielectric Value**	**Tightness Value**
TNF	Tumor necrosis factor	126	2277.08	0.81
ALB	Albumin	122	3027.44	0.79
IL1B	Interleukin-1 betaumin	107	963.72	0.74
JUN	Transcription factor Jun	93	645.04	0.7
SRC	Proto-oncogene tyrosine-protein kinase Src	91	1157.62	0.69
MMP9	Matrix metalloproteinase-9	88	592.87	0.68
CASP3	Caspase-3	85	458.29	0.67
EGFR	Epidermal growth factor receptorumin	85	716.14	0.67
IL2	Interleukin-2	84	366.5	0.67
PTGS2	Prostaglandin G/H synthase 2	77	451.13	0.65
STAT1	Signal transducer and activator of transcription 1-alpha/beta	73	166.5	0.64
PPARG	AlPeroxisome proliferator-activated receptor gamma	72	414.26	0.64
ANXA5	Annexin A5	64	169.2	0.61
MAPK14	Mitogen-activated protein kinase 14	55	79.62	0.6
Mean	-	87	820.39	0.68

**Table 5:** Molecular docking results.

**Gene Symbol**	**PDB ID**	**Total Score**	**Collision Value**	**Polarity**	**CSCORE**
ALB	1HK4	6.12	-1.41	2.18	2
CASP3	3DEK	4.99	-0.99	1.73	2
EGFR	5XDK	6.51	-1.39	2.30	4
IL1B	6Y8M	4.46	-0.71	1.31	5
IL2	1M48	6.30	-0.94	2.25	2
JUN	6Y3V	4.59	-1.45	4.82	5
MMP9	5I12	7.13	-2.43	3.41	4
PTGS2	2FVJ	4.46	-2.95	0.74	4
SRC	4F5B	4.74	-1.61	1.37	3
TLR2	2Z80	2.72	-0.90	2.19	5
TLR3	3ULU	4.73	-0.70	2.69	5
TLR4	3UL7	4.12	-0.95	1.41	5
TNF	5UUI	4.29	-1.00	3.71	4

## Data Availability

The data that support the findings of the article are available in the PubChem, SwissADME, SwissTargetPrediction, PharmMapper, DrugBank, TargetNet, BATMAN-TCM, GeneCards, PHARMGKB, DrugBank, OMIM, DisGeNET databases and all of our public databases.

## LIST OF ABBREVIATIONS

BP: Biological Processes
CC: Cellular Components
ESRD: End-stage Renal Disease
IgAN: IgA Nephropathy
MF: Molecular Function
RAAS: Renin-angiotensin-aldosterone System
SLE: Systemic Lupus Erythematosus
